# Quercetin Mitigates Lysophosphatidylcholine (LPC)-Induced Neutrophil Extracellular Traps (NETs) Formation through Inhibiting the P2X7R/P38MAPK/NOX2 Pathway

**DOI:** 10.3390/ijms25179411

**Published:** 2024-08-30

**Authors:** Si Liu, Yan Wang, Linyao Ying, Hao Li, Keyi Zhang, Na Liang, Gang Luo, Lin Xiao

**Affiliations:** Xiangya School of Public Health, Central South University, Changsha 410013, China; 226911029@csu.edu.cn (S.L.); estherwyan@csu.edu.cn (Y.W.); 226912059@csu.edu.cn (L.Y.); 236912060@csu.edu.cn (H.L.); zhangky@csu.edu.cn (K.Z.); 236912047@csu.edu.cn (N.L.)

**Keywords:** neutrophil extracellular traps, lysophosphatidylcholine, quercetin, P2X7R, P38MAPK, NOX2

## Abstract

Neutrophil extracellular traps (NETs) are three-dimensional reticular structures that release chromatin and cellular contents extracellularly upon neutrophil activation. As a novel effector mechanism of neutrophils, NETs possess the capacity to amplify localized inflammation and have been demonstrated to contribute to the exacerbation of various inflammatory diseases, including cardiovascular diseases and tumors. It is suggested that lysophosphatidylcholine (LPC), as the primary active component of oxidized low-density lipoprotein, represents a significant risk factor for various inflammatory diseases, such as cardiovascular diseases and neurodegenerative diseases. However, the specific mechanism of NETs formation induced by LPC remains unclear. Quercetin has garnered considerable attention due to its anti-inflammatory properties, serving as a prevalent flavonoid in daily diet. However, little is currently known about the underlying mechanisms by which quercetin inhibits NETs formation and alleviates associated diseases. In our study, we utilized LPC-treated primary rat neutrophils to establish an in vitro model of NETs formation, which was subsequently subjected to treatment with a combination of quercetin or relevant inhibitors/activators. Compared to the control group, the markers of NETs and the expression of P2X7R/P38MAPK/NOX2 pathway-associated proteins were significantly increased in cells treated with LPC alone. Quercetin intervention decreased the LPC-induced upregulation of the P2X7R/P38MAPK/NOX2 pathway and effectively reduced the expression of NETs markers. The results obtained using a P2X7R antagonist/activator and P38MAPK inhibitor/activator support these findings. In summary, quercetin reversed the upregulation of the LPC-induced P2X7R/P38MAPK/NOX2 pathway, further mitigating NETs formation. Our study investigated the potential mechanism of LPC-induced NETs formation, elucidated the inhibitory effect of quercetin on NETs formation, and offered new insights into the anti-inflammatory properties of quercetin.

## 1. Introduction

Neutrophils serve as crucial non-specific immune cells, constituting the frontline of the organism’s immune defense. During inflammation, neutrophils respond to chemokines by migrating from circulation to the site of inflammation to defend against pathogens [1]. In 2004, Brinkmann et al. identified a novel effector mechanism in neutrophils and named them neutrophil extracellular traps (NETs) based on their morphological characteristics [2]. The discovery of NETs has significantly advanced research into the role of neutrophils in various inflammatory diseases.

NETs are three-dimensional reticular structures that release chromatin and cellular contents extracellularly upon neutrophil activation. They contain histones and a variety of granule-derived proteins on their nucleic acids, such as myeloperoxidase (MPO), elastase, and citrullinated histone 3 (H3cit). The quantification of NETs formation now commonly involves the detection of cell-free DNA (cf-DNA), MPO-DNA complexes, and the expression of H3cit and MPO [3]. Physiologically, moderate formation of NETs enables neutrophils to capture pathogens, effectively regulating the level of inflammation [4]. However, the major components in NETs possess immunomodulatory properties and serve as the underlying mechanism for amplifying localized inflammatory processes. Excessive production and impaired clearance of NETs lead to tissue damage and pathological conditions [5]. It has been confirmed that NETs are involved in almost all stages of many inflammatory diseases such as atherosclerosis, non-alcoholic fatty liver disease, and rheumatoid arthritis [6,7,8]. Exploring the regulatory mechanism of NETs formation is crucial for inhibiting inflammation and alleviating the progression of inflammatory diseases.

Oxidized low-density lipoprotein (ox-LDL) has been consistently identified as an independent risk factor for a range of acute and chronic inflammatory diseases [9]. It has been shown that ox-LDL possesses antigenic properties, activating both innate and adaptive immune responses, which then lead to inflammatory secretions [10]. As the primary active component of ox-LDL, Lysophosphatidylcholine (LPC) has been demonstrated to be a significant contributing factor for various diseases. It is noteworthy that nearly half of the total phosphatidylcholine in low-density lipoprotein is converted to lysophosphatidylcholine (LPC) during the formation of ox-LDL, which results in more than 30 times as much LPC in ox-LDL compared to the original low-density lipoprotein [11]. Higher levels of LPC in the liver disrupt the integrity of hepatocyte mitochondria and inhibit the expression of genes related to fatty acid oxidation. In atherosclerosis, LPC stimulates the release of inflammatory chemokines from endothelial cells that impair arterial elasticity [12]. Furthermore, LPC also acts on neutrophils by promoting the translocation of azurophilic granules to enhance neutrophil immunoreactivity [13]. However, the NETs formation induced by LPC and its underlying mechanisms remain unknown.

The purinergic ligand-gated ion channel 7 receptor (P2X7R) is a non-selective cation channel. It is highly expressed in neutrophils, smooth muscle cells, and endothelial cells, with abundant pathophysiological roles [14]. Recent studies have demonstrated that P2X7R is prominently expressed in macrophages within atherosclerotic plaques and is closely associated with the release of pro-inflammatory cytokines [15]. The inhibition of P2X7R activation reduces inflammatory factor-induced injury to retinal endothelial cells in diabetes [16]. Critically, physiologically relevant concentrations of LPC can enhance P2X7R sensitivity and mediate a range of cellular responses, including increasing the sustained inward calcium ion flux, promoting cell membrane pore formation, and upregulating p44/42MAPK phosphorylation [17]. The upregulation of P2X7R in neutrophils leads to the release of interleukin-1β, thereby exacerbating the inflammatory response in the organism [18]. However, limited research has been dedicated to P2X7R-mediated NETs formation, and it remains unclear whether LPC activates P2X7R in the inflammatory microenvironment to induce NETs.

NADPH oxidases 2 (NOX2) is a multi-subunit electron-translocating membrane protein complex composed of gp91phox, p22phox, p40phox, p47phox, p67phox and Rac1/2/3 [19], initially characterized in detail in neutrophils. NOX2 catalyzes the production of reactive oxygen species (ROS) and is involved in regulating cell growth, adhesion, differentiation, and apoptosis [20]. Targeting NOX2 expression has been reported to reverse tetrachlorobenzoquinone-induced NETs formation [21]. However, it remains to be determined whether NOX2 plays a role in NETs formation induced by LPC. The molecular regulatory mechanism of this process remains to be further elucidated.

P38-mitogen activated protein kinases (P38MAPK) is a crucial protein kinase involved in the stress-related response within the MAPK cascade. The expression of P38MAPK is present in nearly all cell types. When triggered by various stimuli, P38MAPK is activated by phosphorylation and converts the signals into a series of cellular responses [22,23]. Several studies have confirmed the key role of P38MAPK in NETs formation induced by bongkrekic acid and histamine [24,25]. In particular, P2X7R has been demonstrated to be an upstream regulator of P38MAPK [26], and NOX2 is a downstream target of P38MAPK [27]. Therefore, we hypothesized that the P2X7R/P38MAPK/NOX2 pathway plays a pivotal role in LPC-induced NETs formation in inflammatory diseases, such as atherosclerosis, non-alcoholic fatty liver disease, and arthritis.

Quercetin, one of the most abundant and widespread flavonoids in daily diet, is present in various vegetables and fruits including onions, apples, and grapes [28]. A large number of epidemiological and pre-clinical studies provide strong evidence for the pharmacological potential of quercetin in mitigating inflammatory diseases [29]. At the same time, many in vivo and in vitro studies have shown that quercetin effectively exhibits anti-inflammatory properties by reducing the expression of inflammatory factors and inhibiting pathways involved in the inflammatory response [30]. Inflammatory neutrophils can also be regulated by quercetin, which effectively reduces neutrophil aggregation and inhibits degranulation [31]. Importantly, quercetin regulates NETs-related components. This study found that quercetin has been shown to effectively decrease the expression of MPO in intestinal mucosal cells induced by blood reperfusion in rats [32]. Meanwhile, it is a potent inhibitor of elastase release and inhibits the catalytic activity of elastase [33]. However, whether quercetin regulates the process of NETs formation and its underlying molecular mechanisms need to be further investigated.

Our study used LPC-induced primary rat neutrophils to establish an in vitro model of NETs formation. Based on these observations, it aims to explore the role of LPC in NETs formation and its potential mechanism, as well as the regulatory role of quercetin.

## 2. Result

### 2.1. LPC Induces NETs Formation in Primary Rat Neutrophils

**Figure 1:**
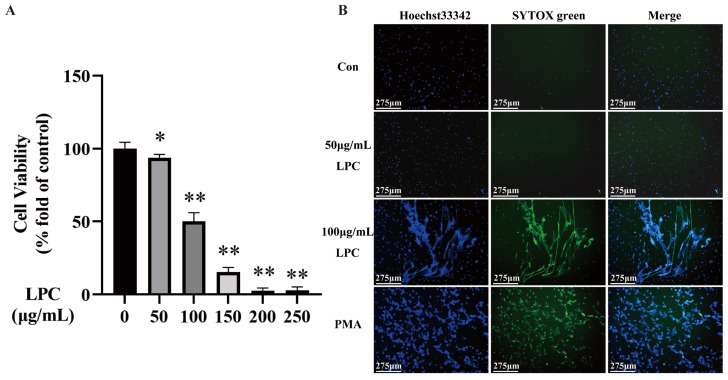
Selecting the proper dose for LPC. (**A**) The effect of LPC on the cell viability of neutrophils. Data are expressed as mean ± SD (n = 4). * *p* < 0.05, ** *p* < 0.01 vs. the control group. (**B**) Live cell imaging of NETs formation induced by LPC and PMA (200 nmol/L). SYTOX green is a cell-impermeable dye that stains extracellular DNA. Hoechst 33342 is a cell-permeable dye that stains both intracellular and extracellular DNA, scale bar = 275 μm.

The peripheral blood of rats was collected, and the primary neutrophils were isolated using a kit. Firstly, to establish a model of NETs, the neutrophils were treated with different concentrations of LPC (0, 50, 100, 150, 200, 250 µg/mL) for 3 h. The CCK-8 assay showed that LPC decreased the viability of neutrophils in a dose-dependent manner. When the treatment concentration of LPC was 100 µg/mL, the cell viability was 50.15% (Figure 1A). Therefore, our study proceeded to investigate the proper concentrations of LPC within the range of 0–100 μg/mL. The formation of NETs can be observed as an extracellular DNA filament structure interwoven into a net [34].Live cell imaging was conducted to observe NETs formation in neutrophils treated with LPC (0, 50, 100 μg/mL) for 3 h (Figure 1B). PMA, the classical inducer of NETs, was used as the positive control. In the control and 50 μg/mL LPC group, no extracellular filamentous or reticulated DNA was observed. However, there were obvious extracellular filamentous or reticulated DNA in the 100 μg/mL LPC group, similar to the PMA group. Based on these results [35], a concentration of 100 µg/mL LPC was selected for subsequent experiments.

**Figure 2:**
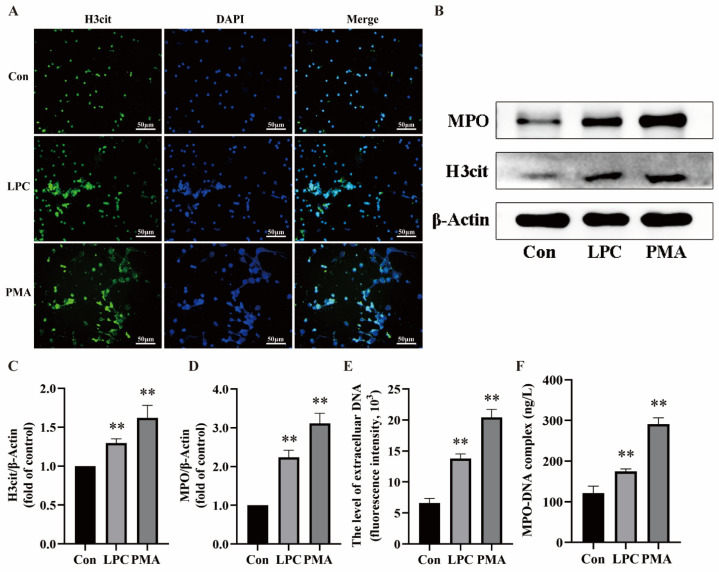
LPC induces NETs formation. (**A**) Cells were treated by RPMI1640, LPC (100 μg/mL), or PMA (200 nmol/L) for 3 h. The formation of NETs was identified by labeling with H3cit and observed by fluorescence microscopy (scale bar, 50 µm). (**B**) Representative immunoblots probed for H3cit, MPO, and β-Actin are shown. (**C**,**D**) Quantitative densitometric analyses of H3cit and MPO normalized to β-Actin (**E**) The supernatant of treated neutrophils was stained with SYTOX green and extracellular DNA was detected using a multimodal reader. (**F**) The level of NETs marker MPO-DNA in the supernatant was detected by ELISA. Data are expressed as mean ± SD (n = 3). ** *p* < 0.01 vs. the control group.

In order to validate the establishment of the in vitro model, our study employed a range of methods to confirm NETs formation. NETs are composed of DNA, H3cit, and MPO. The extracellular release and increased expression of H3cit and MPO indicate NETs formation [36]. Neutrophils were treated with RPMI1640, LPC (100 μg/mL), or PMA (200 nmol/L) for 3 h, respectively. Under a fluorescence microscope, we observed obvious H3cit-attached reticulation in the 100 μg/mL LPC group and the PMA group, but not in the control group (Figure 2A). Western blot analysis was utilized to identify the characteristic proteins of NETs, including H3cit and MPO. The results showed that both 100 μg/mL LPC and PMA treatment increased the expression of H3cit and MPO compared to the control group (Figure 2B–D). Furthermore, NETs were quantified by using SYTOX green staining for cf-DNA, a crucial constituent of NETs. The fluorescence intensity in the 100 μg/mL LPC and PMA groups was higher than in the control group (*p* < 0.01) (Figure 2E). Next, we also conducted an ELISA for MPO-DNA complexes. MPO-DNA is also a characteristic component of NETs. The results showed that the treatment of cells with 100 μg/mL LPC and PMA increased the MPO-DNA level in the supernatant (Figure 2F). These results suggested that 100 µg/mL LPC was the proper concentration to induce NETs in vitro.

### 2.2. Quercetin Inhibits LPC-Induced NETs Formation

**Figure 3:**
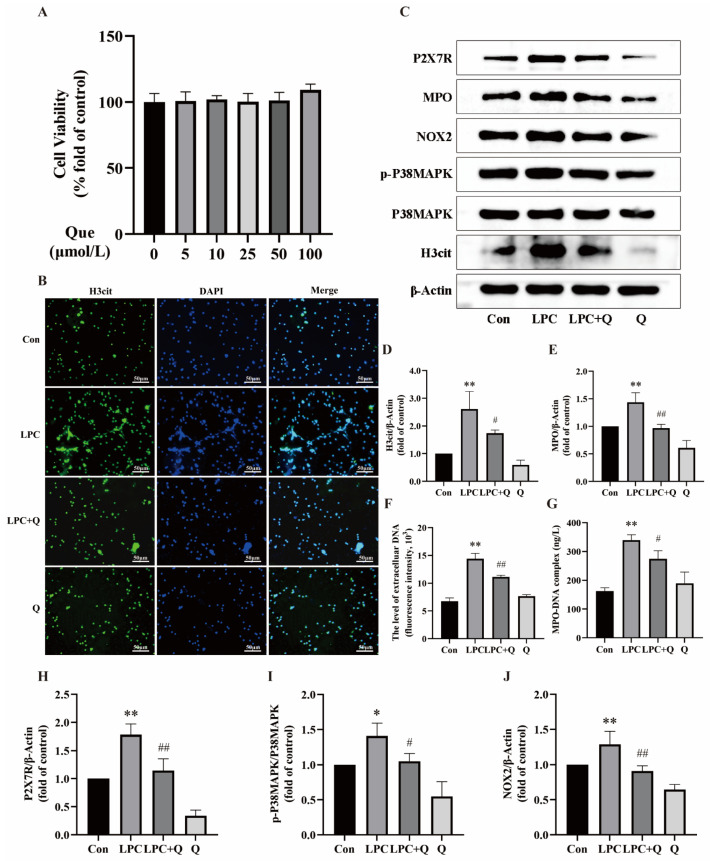
Quercetin inhibits LPC-induced NETs formation. (**A**) The effect of quercetin on the cell viability of neutrophils (mean ± SD, n = 4). (**B**) Cells were treated by RPMI1640, LPC (100 μg/mL), LPC + Q (25 μmol/L), or Q for 3 h. The formation of NETs was identified by labeling with H3cit and observed by fluorescence microscopy (scale bar, 50 µm). (**C**) Representative immunoblots probed for H3cit, P38MAPK, p-P38MAPK, NOX2, MPO, P2X7R, and β-Actin are shown. (**D**,**E**) Quantitative densitometric analyses of H3cit and MPO normalized to β-Actin. (**F**) The supernatant of treated neutrophils was stained with SYTOX green and extracellular DNA was detected using a multimodal reader. (**G**) The level of NETs marker MPO-DNA in the supernatant was detected by ELISA. (**H**–**J**) The ratio of p-P38MAPK/P38MAPK and quantitative densitometric analyses of P2X7R and NOX2 normalized to β-Actin. Data are expressed as mean ± SD (n = 3). * *p* < 0.05, ** *p* < 0.01 vs. the control group. ^#^ *p* < 0.05, ^##^ *p* < 0.01 vs. the LPC group.

In order to explore the effect of quercetin on LPC-induced NETs formation, neutrophils were treated with 100 µg/mL LPC alone or combined with 25 µmol/L quercetin for 3 h. The CCK-8 assay showed that quercetin was non-toxic to neutrophils below 100 μmol/L (Figure 3A). Based on previous literature [37] and the CCK-8 results, a concentration of 25 µmol/L quercetin was selected for subsequent experiments. Immunofluorescence was employed for qualitative analysis of NETs formation. Although LPC induced a distinct extracellular reticulated structure, no specific reticulation was observed in the LPC + quercetin-treated group and only a few short filaments were present outside individual cells. Treatment with quercetin alone did not result in any difference from the control group (Figure 3B). Western blot analysis revealed that quercetin treatment suppressed the elevated levels of H3cit and MPO expression induced by LPC (Figure 3C–E). The results of cf-DNA and MPO-DNA assays also demonstrated that quercetin treatment led to a significant reduction in cf-DNA release and MPO-DNA levels (Figure 3F,G). We suggest that quercetin is a potent inhibitor of LPC-induced NETs formation.

Next, we aimed to further explore the molecular mechanism of LPC-induced NETs formation and the effect of quercetin. The results showed that LPC treatment increased the phosphorylation level of p38MAPK and the expression of P2X7R and NOX2. However, the expression of P2X7R and NOX2, as well as p38MAPK phosphorylation, were all down-regulated following combined treatment with LPC and quercetin compared to the LPC group (Figure 3C,H–J). These findings suggest that P2X7R, P38MAPK, and NOX2 play an important role in the process of LPC-induced NETs formation and the inhibitory effect of quercetin on NETs.

### 2.3. LPC-Induced NETs Formation Is Mediated by P2X7R Up-Regulation

**Figure 4:**
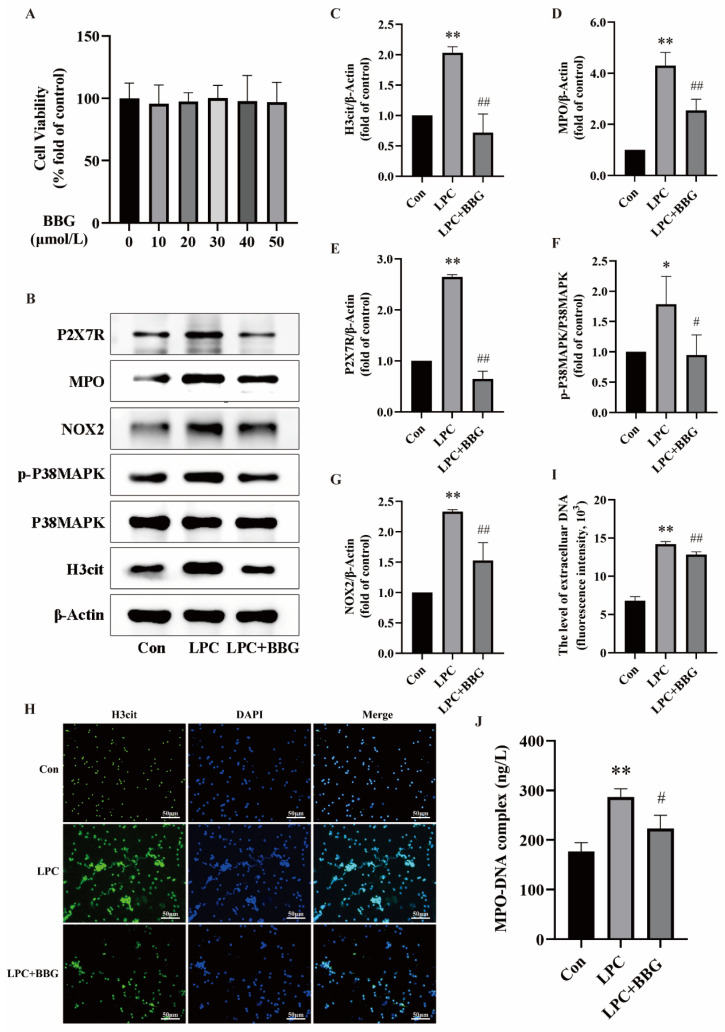
LPC-induced NETs formation depends on up-regulation of P2X7R expression. (**A**) The effect of BBG on the cell viability of neutrophils (mean ± SD, n = 5). (**B**) Cells were treated by RPMI1640, LPC (100 μg/mL), or LPC + BBG (20 μmol/L) for 3 h. Representative immunoblots probed for H3cit, P38MAPK, p-P38MAPK, NOX2, MPO, P2X7R, and β-Actin are shown. (**C**–**G**) The ratio of p-P38MAPK/P38MAPK and quantitative densitometric analyses of H3cit, MPO, P2X7R, and NOX2 normalized to β-Actin. (**H**) The formation of NETs was identified by labeling with H3cit and observed by fluorescence microscopy (scale bar, 50 µm). (**I**) The supernatant of treated neutrophils was stained with SYTOX green and extracellular DNA was detected using a multimodal reader. (**J**) The level of NETs marker MPO-DNA in the supernatant was detected by ELISA. Data are expressed as mean ± SD (n = 3). * *p* < 0.05, ** *p* < 0.01 vs. the control group. ^#^ *p* < 0.05, ^##^ *p* < 0.01 vs. the LPC group.

To confirm whether LPC could trigger NETs formation through P2X7R, neutrophils were treated with 100 µg/mL LPC alone or combined with 20 µmol/L BBG (BBG, a P2X7R antagonist, exhibits at least 1000 times more potency in blocking P2X7R compared to other P2X family members [38]) for 3 h. The NETs formation and the expression of P2X7R-, P38MAPK-, and NOX2-related proteins were then observed. Firstly, the CCK-8 assay showed that BBG was noncytotoxic to neutrophils below the concentration of 50 μmol/L (Figure 4A). Based on previous literature and the results of the CCK-8, a concentration of 20 µmol/L BBG was chosen for subsequent experiments [39]. Western blot analysis showed that the inhibition of P2X7R reduced the expression of H3cit and MPO compared to the LPC group. Additionally, the phosphorylation of P38MAPK and the expression of NOX2 were also significantly decreased (Figure 4B–G). Under the fluorescence microscope, the extracellular reticulated structure of H3cit and DNA co-localization was decreased after the addition of BBG (Figure 4H). The quantification of NETs using cf-DNA and MPO-DNA with the multimode reader revealed a decrease in the levels of cf-DNA and MPO-DNA in the LPC + BBG group compared to the LPC group (Figure 4I,J). The results suggest that the NETs formation by LPC was mediated by the increased expression of P2X7R. P38MAPK and NOX2, downstream targets of P2X7R, play a critical role in LPC-induced NETs formation.

### 2.4. LPC-Induced NETs Formation Is Associated with the P2X7R/P38MAPK/NOX2 Pathway

**Figure 5:**
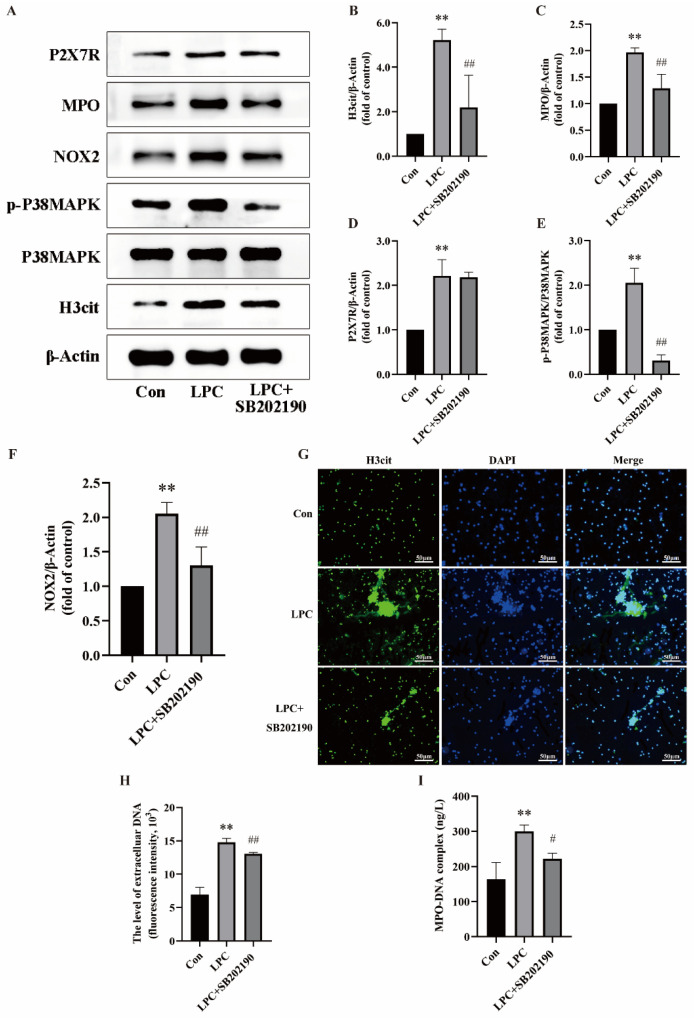
LPC-induced NETs formation is related to the P2X7R/P38MAPK/NOX2 signal pathway. (**A**) Cells were treated by RPMI1640, LPC (100  μg/mL), or LPC + SB202190 (10 μmol/L) for 3 h. Representative immunoblots probed for H3cit, P38MAPK, p-P38MAPK, NOX2, MPO, P2X7R, and β-Actin are shown. (**B**–**F**) The ratio of p-P38MAPK/P38MAPK and quantitative densitometric analyses of H3cit, MPO, P2X7R, and NOX2 normalized to β-Actin. (**G**) The formation of NETs was identified by labeling with H3cit and observed by fluorescence microscopy (scale bar, 50 µm). (**H**) The supernatant of treated neutrophils was stained with SYTOX green and extracellular DNA was detected using a multimodal reader. (**I**) The level of NETs marker MPO-DNA in the supernatant was detected by ELISA. Data are expressed as mean ± SD (n = 3). ** *p* < 0.01 vs. the control group. ^#^ *p* < 0.05, ^##^ *p* < 0.01 vs. the LPC group.

The findings have preliminarily confirmed the association of LPC-induced NETs formation with P2X7R, P38MAPK, and NOX2. However, the relationship between P2X7R, P38MAPK, and NOX2 has not been fully clarified. Our study was further validated by treating neutrophils with SB202190 (10 μmol/L, a P38MAPK inhibitor) to confirm the pivotal role of P38MAPK in LPC-induced NETs formation. The dose of SB202190 is based on the literature [40]. Compared with the LPC-treated group, the expression of H3cit, MPO, and NOX2 were decreased while the expression level of P2X7R did not change in the LPC + SB202190 group (Figure 5A–F). The immunofluorescence findings demonstrated prominent extracellular reticular structures in the LPC group. The co-localization of H3cit and DNA in extracellular reticulated structures was decreased in the LPC + SB202190 co-treated group compared to the LPC group (Figure 5G). Furthermore, there was a decrease in the levels of cf-DNA and MPO-DNA after suppressing the phosphorylation of P38MAPK (Figure 5H,I). These findings demonstrate that NETs formation triggered by LPC is mediated through P38MAPK phosphorylation. The P2X7R/P38MAPK/NOX2 signaling pathway is activated by LPC to induce NETs formation.

### 2.5. Quercetin Inhibits LPC-Induced NETs Formation by Regulating P2X7R

**Figure 6:**
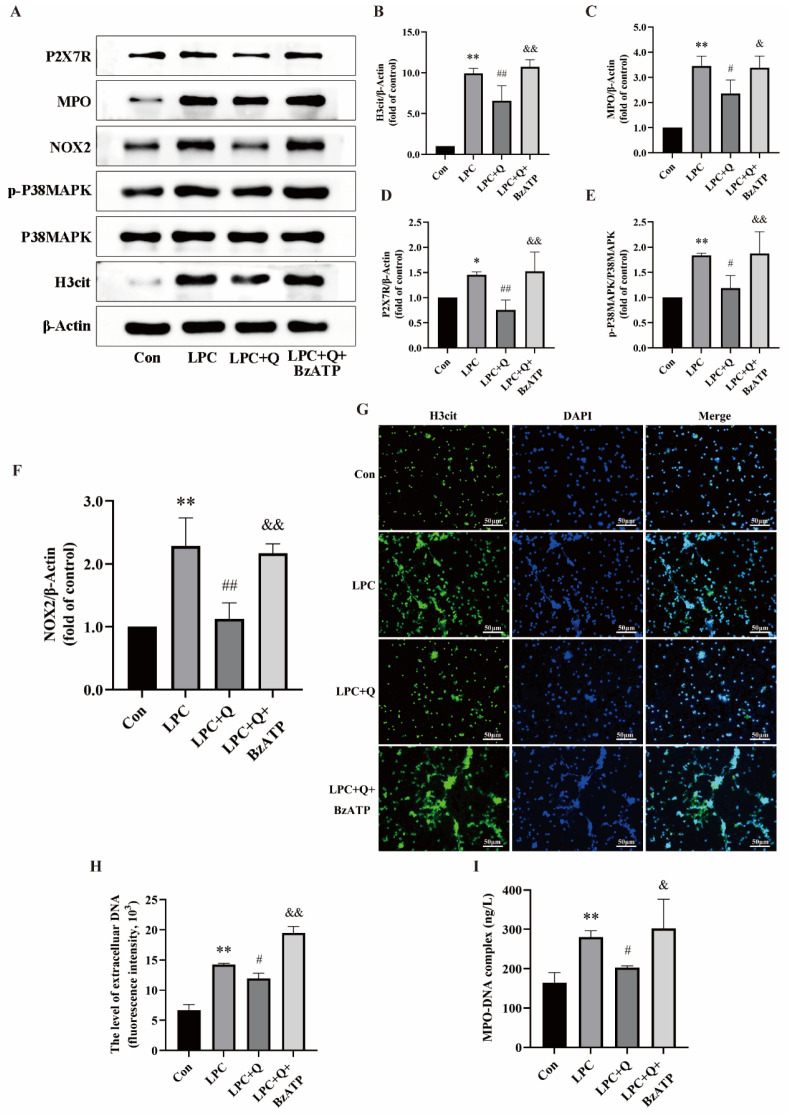
Quercetin inhibits LPC-induced NETs formation by regulating P2X7R. (**A**) Cells were treated by RPMI1640, LPC (100  μg/mL), LPC + Q (25 μmol/L), or LPC + Q + BzATP (100 µmol/L) for 3 h. Representative immunoblots probed for H3cit, P38MAPK, p-P38MAPK, NOX2, MPO, P2X7R, and β-Actin are shown. (**B**–**F**) The ratio of p-P38MAPK/P38MAPK and quantitative densitometric analyses of H3cit, MPO, P2X7R, and NOX2 normalized to β-Actin. (**G**) The formation of NETs was identified by labeling with H3cit and observed by fluorescence microscopy (scale bar, 50 µm). (**H**) The supernatant of treated neutrophils was stained with SYTOX green and extracellular DNA was detected using a multimodal reader. (**I**) The level of NETs marker MPO-DNA in the supernatant was detected by ELISA. Data are expressed as mean ± SD (n = 3). * *p* < 0.05, ** *p* < 0.01 vs. the control group. ^#^ *p* < 0.05, ^##^ *p* < 0.01 vs. the LPC group. ^&^ *p* < 0.05, ^&&^ *p* < 0.01 vs. the LPC + Q group.

In order to explore the inhibition of NETs formation by quercetin targeting P2X7R, neutrophils were treated with 100 µg/mL LPC alone or combined with 25 µmol/L quercetin or 100 µmol/L BzATP (a P2X7R activator) for 3 h. The NETs formation and the expression of P2X7R-, P38MAPK-, and NOX2-related proteins were then observed. The dose of BzATP is based on the literature [41]. Western blot results showed that quercetin suppressed the augmentations in the phosphorylation levels of p38MAPK and the expression of H3cit, MPO, P2X7R, and NOX2 triggered by LPC, and the P2X7R agonist BzATP reversed the effects of quercetin (Figure 6A–F). Under the fluorescence microscope, there was obvious extracellular reticulation in the LPC + quercetin + BzATP treatment group compared with the LPC + Q group (Figure 6G). Next, the presence of BzATP can also reverse the decrease in MPO-DNA and cf-DNA levels induced by quercetin (Figure 6H,I). We suggest that quercetin inhibited LPC-induced NETs formation by decreasing the expression of P2X7R.

### 2.6. Quercetin Inhibits LPC-Induced NETs Formation by Regulating the P2X7R/P38MAPK/NOX2 Pathway

**Figure 7:**
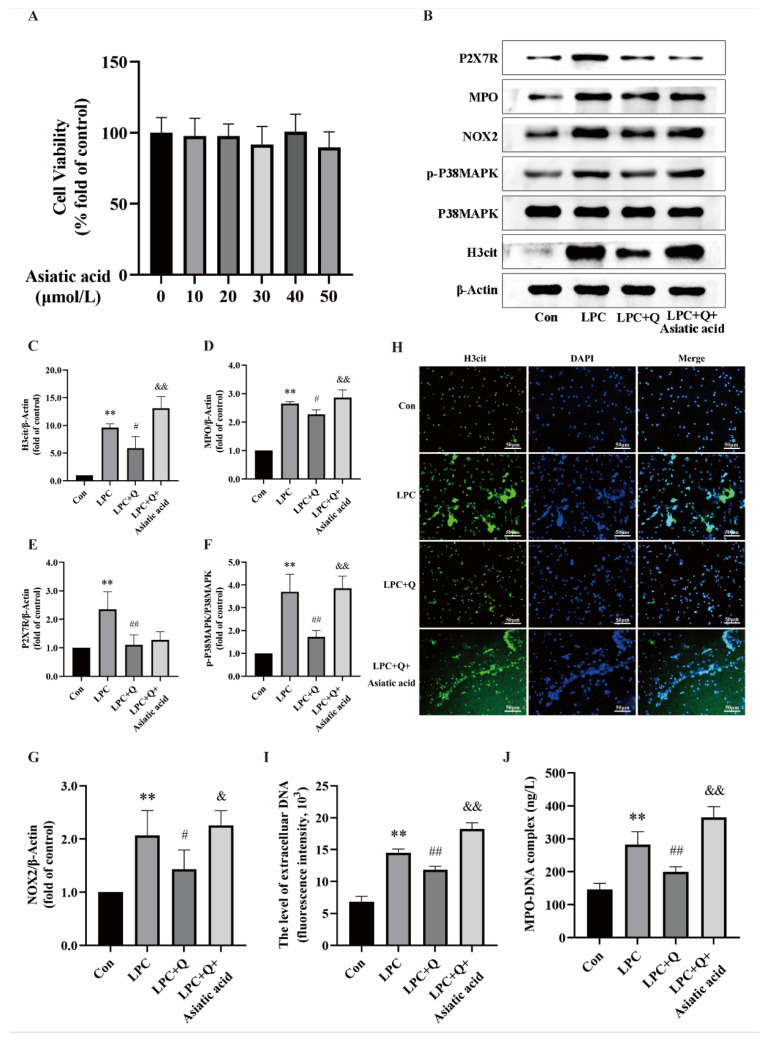
Quercetin inhibits LPC-induced NETs formation by regulating the P2X7R/P38MAPK/NOX2 signal pathway. (**A**) The effect of asiatic acid on the cell viability of neutrophils (mean ± SD, n = 5). (**B**) Cells were treated by RPMI1640, LPC (100 μg/mL), LPC + Q (25 μmol/L), or LPC + Q + asiatic acid (20 µmol/L) for 3 h. Representative immunoblots probed for H3cit, P38MAPK, p-P38MAPK, NOX2, MPO, P2X7R, and β-Actin are shown. (**C**–**G**) The ratio of p-P38MAPK/P38MAPK and quantitative densitometric analyses of H3cit, MPO, P2X7R, and NOX2 normalized to β-Actin. (**H**) The formation of NETs was identified by labeling with H3cit and observed by fluorescence microscopy (scale bar, 50 µm). (**I**) The supernatant of treated neutrophils was stained with SYTOX green and extracellular DNA was detected using a multimodal reader. (**J**) The level of NETs marker MPO-DNA in the supernatant was detected by ELISA. Data are expressed as mean ± SD (n = 3). ** *p* < 0.01 vs. the control group. ^#^ *p* < 0.05, ^##^ *p* < 0.01 vs. the LPC group. ^&^ *p* < 0.05, ^&&^ *p* < 0.01 vs. the LPC + Q group.

The findings have confirmed that the activated P38MAPK/NOX2 pathway is the P2X7R downstream pathway that promotes LPC-induced NETs formation. Further studies are required to confirm whether the inhibitory effect of quercetin on NETs formation is associated with the P38MAPK/NOX2 pathway. Neutrophils were treated with 100 µg/mL LPC alone or combined with 25 µmol/L quercetin or 20 µmol/L asiatic acid (a P38MAPK activator) for 3 h. The CCK-8 assay showed that asiatic acid was noncytotoxic to neutrophils below the concentration of 50 μmol/L (Figure 7A). Based on previous literature and the results of the CCK-8, a concentration of 20 µmol/L asiatic acid was chosen for subsequent experiments [42]. Western blot results showed that quercetin suppressed the augmentations in the phosphorylation levels of p38MAPK and the expression of H3cit, MPO, P2X7R, and NOX2 triggered by LPC. However, compared with the LPC + Q group, the expression of H3cit, MPO, and NOX2 were increased while the expression level of P2X7R did not change in the LPC + Q + asiatic acid group (Figure 7B–G). Under the fluorescence microscope, the extracellular reticulated structure of H3cit and DNA co-localization was increased after the addition of asiatic acid (Figure 7H). The quantification of NETs using cf-DNA and MPO-DNA with the multimode reader revealed a significant increase in the levels of cf-DNA and MPO-DNA in the LPC + Q + asiatic acid group compared to the LPC + Q group (Figure 7I,J). The results show that asiatic acid completely reversed the inhibition of quercetin. This further confirms that the effect of quercetin on inhibiting LPC-induced NETs is dependent on the regulation of the P2X7R/P38MAPK/NOX2 pathway.

## 3. Discussion

Neutrophils, as the main inflammatory cells in organisms, are not only involved in the development of various types of inflammatory diseases; their count also gives a better indication of severity of the disease [43]. However, the short lifespan, rapid renewal, and phenotypic plasticity of neutrophils have posed a longstanding challenge in exploring their pathophysiological role [44]. There is no doubt that the discovery of NETs provides a new perspective for exploring the crucial role of neutrophils in inflammatory diseases. As a unique inflammatory mediator, NETs facilitate monocyte infiltration, enhance the inflammatory response, and expedite the advancement of non-alcoholic fatty liver disease [45]. In inflammatory bowel disease, NETs disrupt the barrier of intestinal epithelial cells and increase disease activity [46]. In addition, NETs exert direct cytotoxic effects by inducing lysis of smooth muscle cell membranes, impacting the stability of atherosclerotic plaques [47]. Therefore, it is essential to suppress NETs formation and explore its underlying mechanisms in order to alleviate the progression of inflammatory diseases.

The formation of NETs is dependent on stimulation by endogenous or exogenous inducers. LPC, a substance with various immunologically active functions, has been demonstrated in limited studies to be involved in NETs formation. Awasthi et al. discovered that a low concentration of LPC (30 μg/mL) was capable of inducing NETs formation in human polymorphonuclear neutrophil leukocytes in vitro [35]. On the other hand, LPC (10 µmol/L) also exhibited an additive effect on PMA-induced NETs formation [48]. The findings of our study showed that treatment of primary rat neutrophils with 100 μg/mL LPC for 3 h effectively induced NETs formation, confirming LPC as a potent inducer of NETs once again.

The molecular mechanisms underlying NETs formation are intricate, involving the regulation of receptor–ligand interactions and a cascade of signal pathways. However, little is known about the receptors, ligands, and signal pathways involved in NETs formation. As a crucial cell membrane receptor, P2X7R participates in various immune responses, such as inflammasome activation, induction of inflammatory mediators, and production of ROS [14]. The study of ischemic brain injury has revealed that adenosine triphosphate released from necrotic cells acts as a damage-associated molecular pattern, inducing NETs formation in a P2X7R-dependent manner, thereby exacerbating inflammation and neuronal damage [49]. The activation of PI3K/Akt and NF-ĸB signaling pathways downstream of P2X7R also contributes to NETs formation. Zhu et al. have discovered that Programmed death ligand 1 regulates autophagy through the PI3K/Akt signaling pathway, mitigating excessive NETs formation to alleviate acute respiratory distress syndrome [50]. In the context of hypobaric hypoxia, the upregulation of NF-ĸB was associated with NETs formation and the exacerbation of kidney injury [51]. Our study found that LPC-induced NETs formation depends on up-regulation of P2X7R expression.

Concurrently, increased NOX activity up-regulates intracellular ROS levels, triggers MPO and peptidyl arginine deiminase 4 activation, and leads to the subsequent release of elastase by the neutrophils. The combined activity of MPO, peptidyl arginine deiminase 4, and elastase leads to the citrullination of histones and decondensation of chromatin. Chromatin and cellular contents are released extracellularly through membrane pores or lysis, ultimately contributing to the formation of NETs [52]. It has been found that the capacity of neutrophils to form NETs is notably reduced in NOX2-deficient mice following infection with Streptococcus pneumoniae compared to wild-type mice [53]. *Staphylococcus aureus*-, *Pseudomonas aeruginosa*-, and lipopolysaccharide-induced NETs all rely on the activity of NOX2 [54,55]. Our study identified NOX2, a member of the NOX family, as a potential key protein involved in regulating the LPC-induced formation of NETs.

As a crucial signal molecule regulating cellular responses, P38MAPK plays a role in NETs formation triggered by various endogenous and exogenous substances such as free fatty acids, bongkrekic acid, and histamine [24,25,56]. Particularly, P2X7R has been demonstrated to be an upstream regulator of P38MAPK. Leng et al. found that knockdown of P2X7R inhibits the phosphorylation level of P38MAPK, which mitigates high glucose-induced endothelial cell dysfunction [26]. Furthermore, NOX2 is a downstream target of P38MAPK. Hua et al. discovered that the inhibition of P38MAPK phosphorylation suppresses the up-regulation of NOX2 expression induced by advanced glycation end products, resulting in a vasoprotective effect [27]. Our study suggested that the P2X7R/P38MAPK/NOX2 signal pathway is implicated in the regulation of LPC-induced NETs formation. The treatment of neutrophils with a P2X7R antagonist decreased P38MAPK phosphorylation levels, NOX2 expression, and NETs formation. In the future, it will be interesting to test other recently developed P2X7R antagonists. Similarly, treatment of neutrophils with P38MAPK-specific inhibitors did not significantly alter P2X7R expression, but markedly reduced NOX2 expression and NETs formation.

The regulation and mechanism of quercetin in NETs formation are still emerging. Limited studies have reported the role of quercetin in inhibiting NETs formation and reducing NETs-mediated cytotoxicity and tissue damage. Yuan et al. found that quercetin, a potential therapeutic agent for rheumatoid arthritis, can be targeted to inhibit NETs formation at the site of arthrosis in mice [37]. Pereira et al. confirmed that quercetin mitigates the cytotoxic effects of NETs on alveolar basal cells by inhibiting NETs formation [57]. In our study, quercetin inhibited LPC-induced NETs formation. These studies offer a crucial theoretical foundation for the inhibition of NETs formation by quercetin, mitigating associated inflammatory diseases.

Recent research has indicated that quercetin has the capacity to modulate P2X7R. Zhao et al. reported that quercetin modulates oxidative stress in vivo by inhibiting P2X7R, leading to a reduction in ethanol-induced hepatic steatosis in zebrafish [58]. P38MAPK is also a target of quercetin. Li et al. reported that quercetin has the potential to attenuate myocardial ischemia-induced apoptosis by inhibiting P38MAPK phosphorylation [59]. Notably, quercetin antagonized the high expression of NOX2 in rat myocardial cells, leading to a further reduction in NOX2-mediated mitochondrial damage and oxidative stress [60]. Our study was further validated through adding the specific activators for P2X7R and P38MAPK. The findings suggested that quercetin effectively suppresses the formation of LPC-induced NETs by modulating the P2X7R/P38MAPK/NOX2 signal pathway, thereby exhibiting potent anti-inflammatory properties.

It is noteworthy that our study discovered that the treatment of neutrophils with quercetin alone also resulted in the down-regulation of NETs marker proteins and P2X7R/P38MAPK/NOX2 signaling pathway-related proteins. This phenomenon may be associated with the direct impact of quercetin on proteins in resting state, influencing their expression or assembly [61,62,63,64,65]. The biological effect of quercetin further highlights its potential health-promoting properties.

The present study only explored the mechanisms using inhibitors and agonists, and future studies should confirm the role of this pathway in NETs formation by employing silencing and over-expression of various proteins involved in the P2X7R/P38MAPK/NOX2 signal pathway. In addition, our study lacked in vivo experimental support, which requires further clarification.

## 4. Materials and Methods

### 4.1. Reagents and Antibodies

The following reagents were used: Rat peripheral blood neutrophil isolation kit (HaoYang Biological, Tianjin, China), LPC (GlpBio, Montclair, CA, USA), PMA (APExBIO, Boston, MA, USA), quercetin (purity: 98%, Sigma-Aldrich, St. Louis, MO, USA), BBG (Abmole, Houston, TX, USA), SB202190 (MCE, Monmouth Junction, NJ, USA), BzATP (MCE, Monmouth Junction, NJ, USA), asiatic acid (APExBIO, Boston, MA, USA), Hoechst 33342 (Meilunbio, Dalian, China), SYTOX green (Thermo Fisher Scientific, Waltham, MA, USA), DAPI (Servicebio, Wuhan, China), poly-L-lysine (Beyotime Biotechnology, Shanghai, China). All other chemicals used were of reagent grade and purchased from commercial suppliers. The primary antibodies used for Western blot and immunofluorescence were anti-H3cit (Abcam, Cambridge, UK), anti-MPO (Proteintech, Wuhan, China), anti-P2X7R (Proteintech, Wuhan, China), anti-P38MAPK (Cell Signaling Technology, Danvers, MA, USA), anti-p-P38MAPK (Cell Signaling Technology, Danvers, MA, USA), anti-NOX2 (Proteintech, Wuhan, China), β-Actin (Servicebio, Wuhan, China). Horseradish peroxidase (HRP)-linked anti-rabbit IgG and HRP-linked anti-mouse IgG were purchased from Beyotime Biotechnology. Anti-rabbit-488 secondary antibodies were purchased from Servicebio.

### 4.2. Animals

Sprague-Dawley male rats weighing 500 ± 10 g were provided by Changsha Tianqin Biotechnology Co (XYGW-2020-17). All animal experiments were approved by the Ethics Committee for Experimental Animal Welfare of Central South University (Changsha, China). The animals had free access to food and water in a climate controlled room (specific pathogen free) with alternating light and darkness for a standard 12 ± 2 h (24 ± 1 °C, 50 ± 10% humidity). The animal experiments were conducted in accordance with the guiding principles of the regulations on the Administration of Experimental Animals by State Scientific and Technological Commission.

### 4.3. Primary Rat Neutrophils Isolation

To induce anesthesia, the rats were administered 10% chloral hydrate. A median abdominal incision was made with blunt dissection to fully expose the abdominal aorta. The abdominal aorta was then punctured using a disposable venipuncture needle. The rats’ blood was collected in anticoagulation tubes containing sodium citrate, followed by an operation carried out strictly according to the instructions of the rat peripheral blood neutrophil isolation kit.

### 4.4. Cell Culture and Treatment

The primary rat neutrophils were resuspended in RPMI1640 supplemented with 10% fetal bovine serum, 100 U/mL penicillin, and 100 µg/mL streptomycin, then cultured in a 5% CO_2_ incubator at 37 °C. In order to examine the mechanisms of LPC-induced NETs formation and the intervention effect of quercetin, neutrophils were treated with LPC (100 µg/mL) or with quercetin (25 µmol/L), BBG (20 µmol/L, a P2X7R antagonist), SB202190 (10 µmol/L, a P38MAPK inhibitor), BzATP (100 µmol/L, a P2X7R agonist), and asiatic acid (20 µmol/L, a P38MAPK agonist) for 3 h. Based on the results of cell viability assays and existing studies, proper doses of these compounds were selected.

### 4.5. Cell Viability Assays

The viability of the primary rat neutrophils was measured using Cell Counting Kit-8 (Abbkine Scientific, Wuhan, China) after treatment with LPC (0, 50, 100, 150, 200, 250 μg/mL), quercetin (0, 5, 10, 25, 50, 100 µmol/L), BBG (0, 10, 20, 30, 40, 50 µmol/L) or asiatic acid (0, 10, 20, 30, 40, 50 µmol/L) at different concentrations for 3 h.

### 4.6. Live-Cell Imaging

The neutrophils were seeded on 24-well plates coated with poly-L-lysine and incubated with RPMI1640, phorbol 12-myristate 13-acetate (PMA) (200 nmol/L) or different concentrations of LPC for 3 h at 37 °C in a 5% CO_2_ atmosphere. NETs were detected using the cell-permeable dye Hoechst 33342 (10 μg/mL) and the cell-impermeable dye SYTOX green (100 nmol/L) to label the DNA; they were stained for 20 min and 10 min, respectively, and protected from light. The formation of NETs was observed under a fluorescence microscope.

### 4.7. Immunofluorescence

Isolated neutrophils were seeded into 24-well plates with coverslips coated with poly-l-lysine and incubated with RPMI1640, LPC, PMA, BBG, SB202190, BzATP, asiatic acid, or quercetin for 3 h at 37 °C in a 5% CO_2_ atmosphere. Next, the cells were fixed in 4% paraformaldehyde with permeabilization by 0.5% Triton and blocked with goat serum at 37 °C for 1 h. The coverslips were placed on slides that were labelled with an immunohistochemistry pen. Anti-H3cit primary antibodies were added to the coverslips, and they were stored overnight at 4 °C. This was followed by detection with anti-rabbit-488 secondary antibodies for H3cit visualization at room temperature for 1 h. DAPI was used to stain cell nuclei. The NETs formation was observed by fluorescence microscopy after the addition of anti-fluorescence quencher.

### 4.8. Quantification of cf-DNA

Isolated neutrophils were seeded into 6-well plates with poly-L-lysine coating, then the cells were treated with RPMI1640, LPC, PMA, BBG, SB202190, BzATP, asiatic acid, or quercetin for 3 h at 37 °C with 5% CO_2_. The supernatant in the culture dishes was collected and centrifuged at 1000 rpm for 5 min to remove cellular debris. SYTOX green dye was added (the final concentration was 1 μmol/L) and mixed with the cell supernatant, then dyed for 20 min away from light. After that, the cell supernatant was transferred into a black 96-well plate (200 μL per well). A Multimode reader was used to quantify the level of extracellular DNA, with the excitation light set to 485 nm and the emission light to 528 nm.

### 4.9. MPO-DNA Complexes ELISA

The neutrophils were seeded on 6-well plates coated with poly-L-lysine, then the cells were treated with RPMI1640, LPC, PMA, BBG, SB202190, BzATP, asiatic acid, or quercetin for 3 h at 37 °C with 5% CO_2_. The supernatant in the culture dishes was collected and centrifuged at 1000 rpm for 5 min to remove cellular debris. NETs-associated MPO-DNA complexes were quantified using the rat ELISA kit (FANKEW, Shanghai, China). The quantification operation was strictly in accordance with the instruction manual of the ELISA kit. The OD value at 450 nm was determined and reflects the amount of MPO-DNA.

### 4.10. Western Blot Analysis

Primary rat neutrophils were homogenized and lysed on ice in a RIPA lysis buffer (Servicebio, Wuhan, China) containing the Phosphatase Inhibitor Cocktail (DINGGUO CHANGSHENG Biotechnology, Beijing, China). The protein concentration was measured using a BCA kit (Abbkine Scientific, Wuhan, China). Equal amounts of proteins were separated by SDS-PAGE and transferred onto polyvinylidene difluoride (PVDF, Millipore Corp, Billerica, MA, USA) membranes by electrophoretic transfer. After blocking with milk, the membranes were incubated overnight at 4 °C with the primary antibodies (H3cit, 1:1000; P38MAPK, 1:1000; p-P38MAPK, 1:1000; NOX2, 1:4000; MPO, 1:1000; P2X7R, 1:3000; β-Actin, 1:1000). Next, the membranes were incubated with HRP-conjugated secondary antibodies (anti-rabbit, 1:8000; anti-mouse, 1:10,000). Immunoreactive bands were detected using the ECL kit (BIOPRIMACY, Wuhan, China) and the Western Blotting Detection System. Meanwhile, Image-J 2.14.0 software was applied to quantify the intensity values of the bands. β-Actin was used as the internal control protein.

### 4.11. Statistical Analysis

SPSS 25.0 software was applied to statistically analyze the experimental data. All measures were normality-tested and chi-square-tested. Normal distribution and chi-squared variance data were expressed as mean ± standard deviation ($\overset{-}{x}$ ± s). One-way ANOVA was used for comparisons between multiple groups, and the LSD test was used for comparisons between two groups when the difference between groups was statistically significant. The results were considered statistically significant at *p* < 0.05.

## 5. Conclusions

Our study provides in vitro evidence of the role of LPC in triggering NETs formation and the inhibitory effect of quercetin. Quercetin reversed the upregulation of the LPC-induced P2X7R/P38MAPK/NOX2 pathway, further mitigating the NETs formation. Our study provides new insights into the protective effects of quercetin by focusing on NETs formation through the P2X7R/P38MAPK/NOX2 pathway, and identifies potential targets for protecting against related inflammatory diseases.

## Data Availability

Data are contained within the article.
